# Genome-wide investigation of the TIFY transcription factors in alfalfa (*Medicago sativa* L.): identification, analysis, and expression

**DOI:** 10.1186/s12870-024-05378-w

**Published:** 2024-09-06

**Authors:** Qi Chen, Rui Dai, Shuang Shuang, Yan Zhang, Xiaowei Huo, Fengling Shi, Zhiqiang Zhang

**Affiliations:** 1 Technology Engineering Center of Drought and Cold-Resistant Grass Breeding in North of the National Forestry and Grassland Administration, College of Grassland, Resources and Environment, Inner Mongolia Agricultural University, Hohhot, China; 2 Key Laboratory of Grassland Resources of the Ministry of Education, College of Grassland, Resources and Environment, Inner Mongolia Agricultural University, Hohhot, China

**Keywords:** Alfalfa, *TIFY* gene family, Biotic stress, Abiotic stress, Expression profiles

## Abstract

**Background:**

Alfalfa (*Medicago sativa* L.) is an essential leguminous forage with high nutrition and strong adaptability. The TIFY family is a plant-specific transcription factor identified in many plants. However, few reports have been reported on the phylogenetic analysis and gene expression profiling of *TIFY* family genes in alfalfa.

**Result:**

A total of 84 *TIFY* genes belonging to 4 categories were identified in alfalfa, including 58 *MsJAZs*, 18 *MsZMLs*, 4 *MsTIFYs* and 4 *MsPPDs*, respectively. qRT-PCR data from 8 genes in different tissues revealed that most *MsTIFY* genes were highly expressed in roots. The expression of *MsTIFY14* was up-regulated after different times in both thrips-resistant and susceptible alfalfa after thrips feeding, and the expression of the remaining *MsTIFYs* had a strong correlation with the time of thrips feeding. Different abiotic stresses, including drought, salt, and cold, could induce or inhibit the expression of *MsTIFY* genes to varying degrees. In addition, the eight genes were all significantly up-regulated by JA and/or SA. Interestingly, *MsTIFY77 was* induced considerably by all the biotic, abiotic, or plant hormones (JA or SA) except ABA.

**Conclusion:**

Our study identified members of the *TIFY* gene family in alfalfa and analyzed their structures and possible functions. It laid the foundation for further research on the molecular functions of *TIFYs* in alfalfa.

**Supplementary Information:**

The online version contains supplementary material available at 10.1186/s12870-024-05378-w.

## Introduction

Alfalfa (*Medicago sativa* L.) is one of the most essential plants for the development of animal husbandry and dairy industry [1]. With the expansion of planting area and the improvement of intensive planting, alfalfa planting also faces uncertain factors such as low yield, diseases and insect pests [2, 3]. To resist these problems, researchers have identified multiple defense mechanisms at the molecular level of plants by mobilizing a wide range of stress-response genes [4]. Therefore, for the development of the alfalfa industry, more attention should be paid to identifying critical functional genes of alfalfa and breeding resistant varieties [5].

Transcription factors (TFs) have a unique structure and regulate the transcription process of genes by binding to the regulatory region’s DNA sequence [6–8]. TFs participate in regulating plant growth and development by activating or inhibiting gene expression and play a vital role in the stress response of plants [9, 10]. Initially, this protein was referred to as ZIM (Zinc-finger protein expressed in Inflorescence Meristem) proteins due to their C_2_C_2_-GATA zinc-finger structure. Subsequently, they were renamed the TIFY family, drawing from the conserved core motif TIF[F/Y]XG [11]. According to the conserved domain types, the TIFY family can be divided into four categories: TIFY, ZML, PPD, and JAZ [12]. The TIFY transcription factors play a significant role in the growth and development of plants, stress response, and hormone signal transduction [13]. To date, studies on the *TIFY* gene family have been reported in many plants. A total of 18, 20, 21, 38 and 47 *TIFY* genes were identified and analyzed in *Arabidopsis thaliana* [11], *Oryza sativa* [14], *Byachypodium distachyon* [15], *Soybean* [16] and *Maize* [17]. These reports are favorable resources for the study of the *TIFY* gene family. In rice, overexpression of *OsTIFYs* genes can not only increase grain size through enhanced accumulation of carbohydrates in the stem, but also significantly increase salt and dehydration tolerance [18, 19]. Similarly, overexpression of *TdTIFY11a* can promote wheat germination under salt stress [20]. In addition, many studies have proposed that *TIFY* family members, especially the JAZ subfamily proteins, are critical regulators of the JA signaling pathway [21–24]. Thus, TIFY TFs have major significance in enhancing plant tolerance to various stresses.

However, there have been no reports in terms of the *TIFY* gene family in alfalfa. In this study, 84 genes were screened from the alfalfa *TIFY* gene family, and the conserved motif, phylogenetic analysis, and protein interaction network diagram prediction of the family member were systematically analyzed. Quantitative Real-Time PCR was used to analyze the gene expression of *TIFY* in different tissues (roots, stems, old leaves, tender leaves, flowers, pods, and seeds), biotic stress (thrips), abiotic stresses (drought, salt, and cold) and hormones stresses (JA, SA, and ABA). These findings will provide valuable insights for the functional characterization of *TIFY* genes in alfalfa.

## Result

### Identification and classification of TIFY proteins

**Fig. 1:**
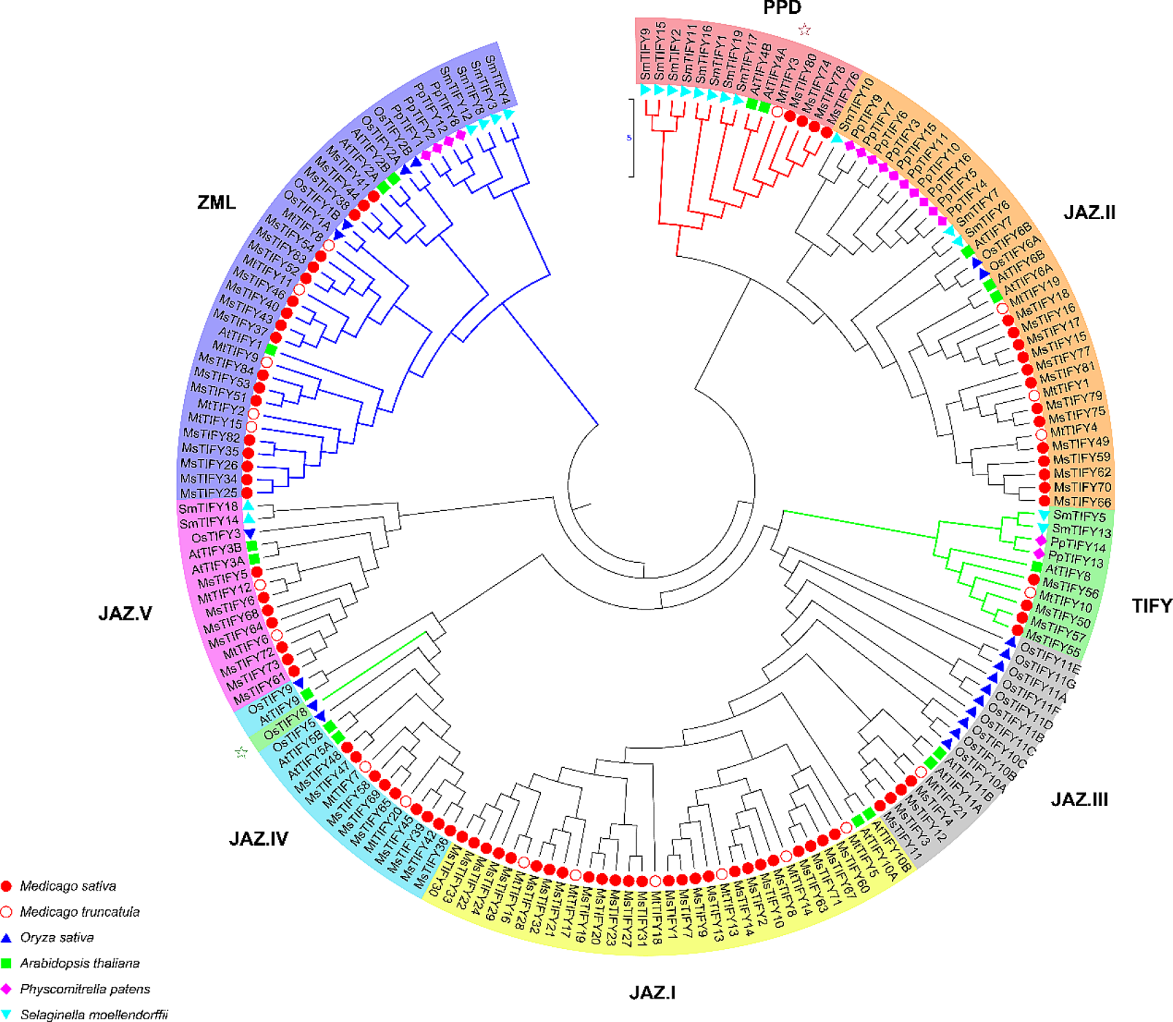
Phylogenetic relationships of *Physcomitrella patens*, *Selaginella moellendorffii*, *Oryza sativa*, *Arabidopsis thaliana*, *Medicago truncatula*, and *Medicago sativa*. Different groups are marked with different colours

According to the results of a BLAST alignment and the HMM, there were 16, 19, 20, 18, 21, and 84 genes were screened from the whole genome of *Physcomitrella patens*, S*elaginella moellendorffii*, *Oryza sativa*,* Arabidopsis thaliana*,* Medicago truncatula* and *Medicago sativa* (Table 1), respectively. The nomenclature of the *TIFY* family genes in *Arabidopsis* and *Oryza sativa* is based on the UniProt database (www.uniprot.org). *Physcomitrella patens*, *Selaginella moellendorffii*, *Medicago truncatula*, and *Medicago sativa TIFY* family genes were based on the order in which the sequences are arranged on the chromosome (Tab.S1). Furthermore, according to the characteristics of the conserved domain, they were classified into four subfamilies, namely TIFY, JAZ, PPD, and ZML (Table 1). In addition, the phylogenetic tree analysis of the above 178 amino acid sequences was carried out, and then we further classified them according to the phylogenetic tree of the JAZ subfamily sequence and the characteristics of the motif sequence, and then divided them into five sublevels of JAZ.I, II, III, IV, and V branch (Fig. 1).

The total number of genes and the number of genes in different subfamilies obtained after analysis and separated by our method are basically consistent with those reported in Bai [12]. Since *Medicago sativa* was tetraploid, while *Medicago truncatula* and other plants were diploid, the number of genes in alfalfa was significantly expanded. 84 *TIFY* genes were identified in total, including 4 *MsTIFYs*, 58 *MsJAZs* (25 class I, 13 class II, 4 class III, 9 class IV, and 7 Class V), 4 *MsPPDs*, and 18 *MsZMLs*. *P. patens* and *O. sativa* did not possess PPD subfamily genes.

The results showed that *TIFY* family genes had low sequence similarity among different species, with an average homology of 46.53%. The *TIFY* subfamily was found in Bryophyta, Lycophyta, monocotyledonous, and dicotyledonous plants. However, in each subfamily, the *TIFY* evolutionary relationships of plants from different species were more similar. For example, the *TIFY* evolutionary relationships of dicotyledonous plants were more clustered in one branch. This suggests that *TIFY* evolved later than the differentiation of bryophyta, Lycophyta, monocotyledon and dicotyledon.

**Table 1:** List of the number of genes of the TIFY family

subfamily	Bryophyta	Lycopodiophyta	Eudicots			Monocots
	*P*. patens	S. moellendorffii	A. thaliana	M. truncatula	M. sativa	O. sativa
TIFY	3	2	1	1	4	1
JAZ	9	8	12	14	58	15
PPD	0	4	2	1	4	0
ZML	4	4	3	5	18	4

### Structure analysis of the *TIFY* gene family in alfalfa

Basic information about the members of the alfalfa *TIFY* gene family was listed in Table S2. The results showed that the full-length coding sequences (CDS), protein length, isoelectric points (pI), molecular weights (Mw), chromosomes and initiation sites are quite different. According to the analysis of chromosome gene sequence length, the most extended *TIFY* family in alfalfa was *MsTIFY30*, with a length of 16,712 bp, and the shortest gene was *MsTIFY20*, with a length of 342 bp. According to the analysis of protein sequence length, it was found that the sequence length of the *TIFY* family in alfalfa was 87 ~ 429aa. The molecular weight is 9.162 KDa～46.23 KDa.

**Fig. 2:**
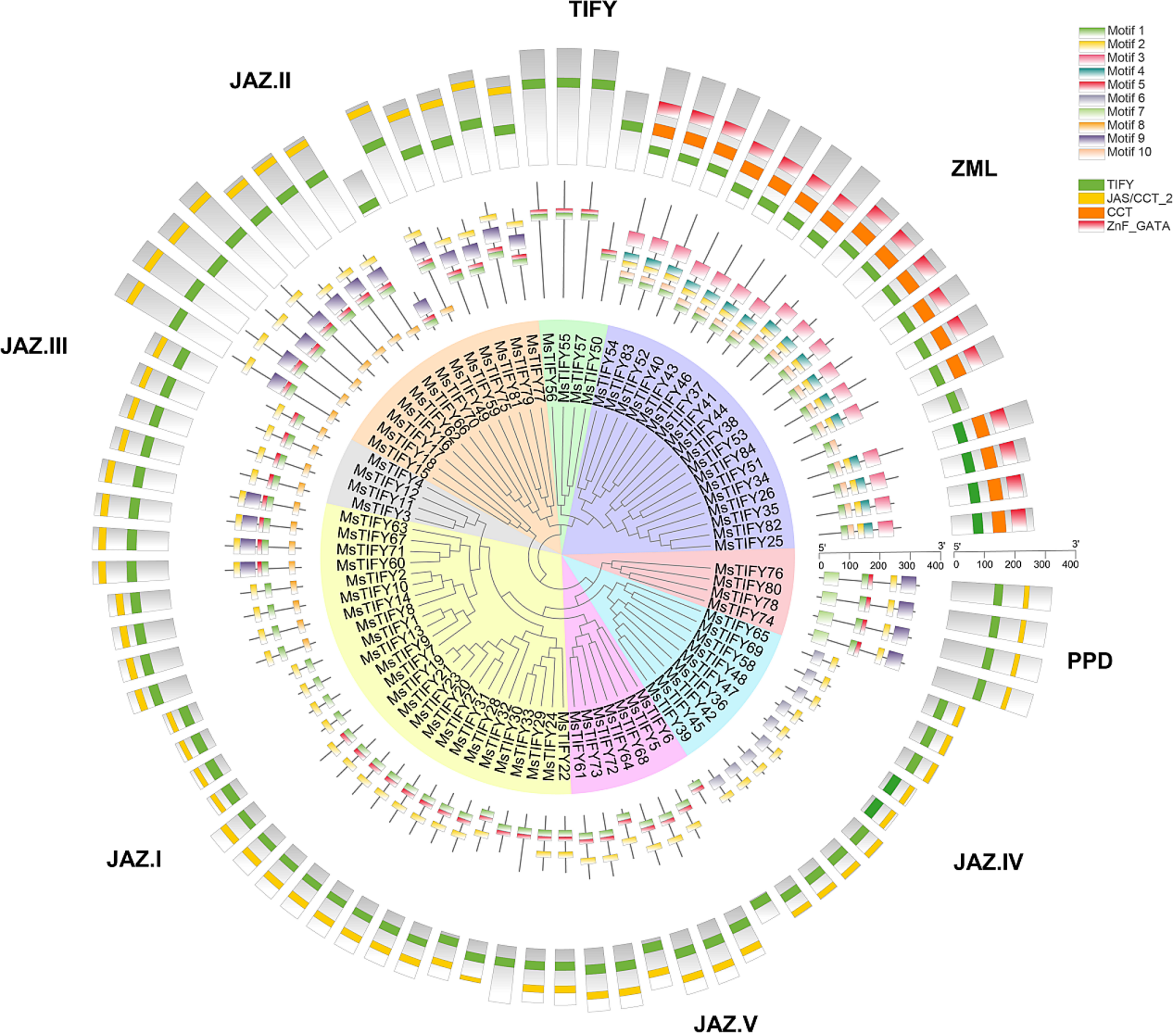
Analysis of genetic structure characteristics of the *TIFY* family phylogenetic tree, motif, and domain genes in alfalfa. The innermost circle is the *TIFY* family evolutionary tree of alfalfa, the middle is motif sequence composition characteristics, and the outer circle is domain gene structure characteristics

The structural characteristics of the *TIFY* family evolutionary tree, motif and domain genes in alfalfa were analyzed (Fig. 2). A total of 10 conserved motifs were detected in the *MsTIFY* genes, which were designated Motifs 1–10, and the members in the same subfamily shared similar conserved motifs. The TIFY subfamily only has the TIFY conserved domain, and the corresponding motif is composed of motif 1 and motif 5 in series. PPD consists of the PPD motif (motif 7), TIFY conserved domain, and JAS/CCT-2, including a sequence of motif 9 at the C-terminal. ZML consists of TIFY, CCT, and GATA domains, and the motif composition is different from other subfamilies, consisting of motif 1, motif 10, motif 2, motif 4, and motif 3. The JAZ subfamily is the largest, with 58 members, and is made up of TIFY conserved domains and JAS/CCT-2. Based on motifs, conserved domain, sequence alignment and phylogenetic tree analysis, the JAZ subfamily was divided into four groups (25 in JAZ I, 13 in JAZ II, 4 in JAZ III, 9 in JAZ IV, and 7 in JAZ V). The presence of subfamily-specific conversed motifs in the subfamily may play a critical role in functional specificity.

### TIFY protein-protein interaction (PPI) network in alfalfa

**Fig. 3:**
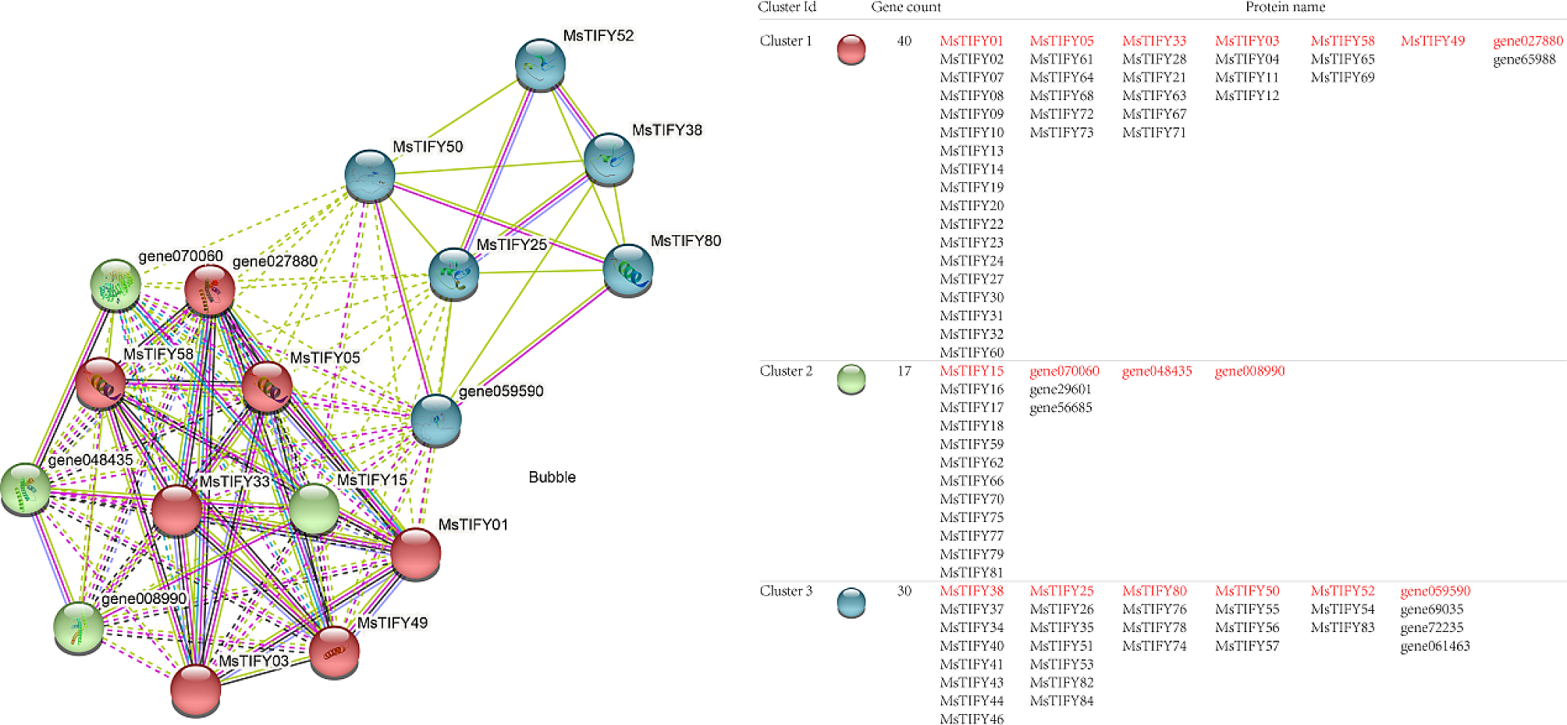
MsTIFY proteins interaction network diagram. Red mark cluster I, green mark cluster 2, and blue mark cluster 3. The right table shows the genes that may be involved in the interaction of the three clusters. The red letters represent the genes identified in the left figure, and the genes below the genes are other homologous genes of this gene in alfalfa

Using protein-protein interactions to connect unknown functional proteins into protein interaction networks will help understand the biological functions of proteins [25, 26]. In this study, *Arabidopsis* was used as a background to predict the potential interacting proteins associated with the protein function of *MsTIFYs* (Tab.S3). A total of 76 *TIFY* family genes found their positions in the interaction network. It was divided into three clusters according to the degree of their interaction with other family genes (Fig. 3). The results showed that the MsTIFY proteins interacted with proteins such as nuclear-localized protein, Jasmonate-zim-domain protein 8, MYC-related transcriptional activator, Coronatine-insensitive protein 1, Jasmonate-zim-domain protein 3, DNA-binding family protein MYC and GATA transcription factor 24. It is speculated that MsTIFY proteins may work synergistically with other proteins in jasmonic acid-mediated plant resistance defense.

### Expression of *MsTIFY* genes in different tissues

**Fig. 4:**
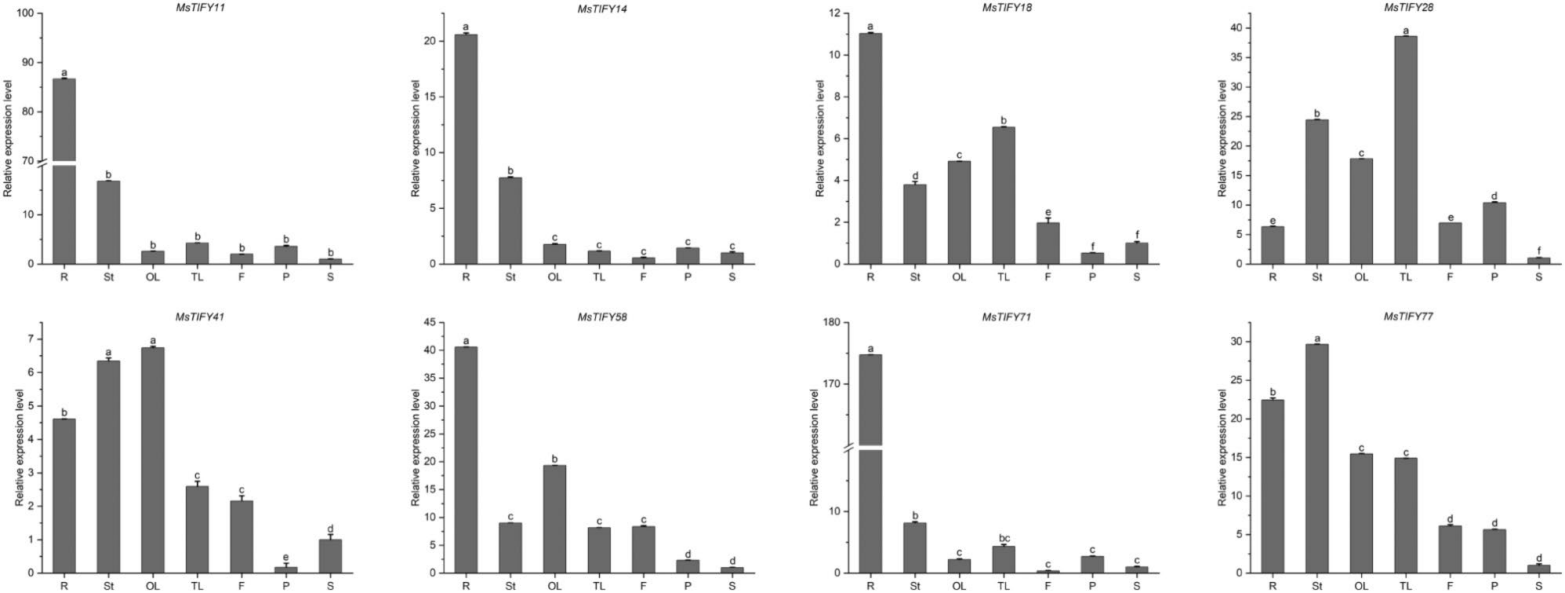
qRT-PCR analysis of the expression patterns of eight *MsTIFY* genes in seven tissues in alfalfa. R: roots; St: stems; OL: old leaves; TL: tender leaves; F: flowers; P: pods; S: seeds. The error bars indicate the standard errors of three biological replicates. Columns with different letters are significantly different(*P* < 0.05)

In order to understand the expression profile of *MsTIFY* genes in different tissues, the expression of 8 *MsTIFY* genes in roots, stems, old leaves, tender leaves, flowers, pods, and seeds were analyzed by qRT-PCR (Fig. 4; Tab.S4). The results showed that *MsTIFY11*,* MsTIFY14*, *MsTIFY18*, *MsTIFY58* and *MsTIFY71* were highly expressed in roots. *MsTIFY77* was preferentially expressed in stems, and *MsTIFY28* was highest expressed in tender leaves. *MsTIFY41* was preferentially expressed in older leaves and highly expressed in roots and stems. The expression level of the flower was relatively high in *MsTIFY28*, *MsTIFY58* and *MsTIFY77*. The results showed that the *MsTIFY* genes had overlapping but spatially varying tissue expression, indicating that they may play different roles in specific tissues.

### Expression of *MsTIFY* genes in response to thrips infection

**Fig. 5:**
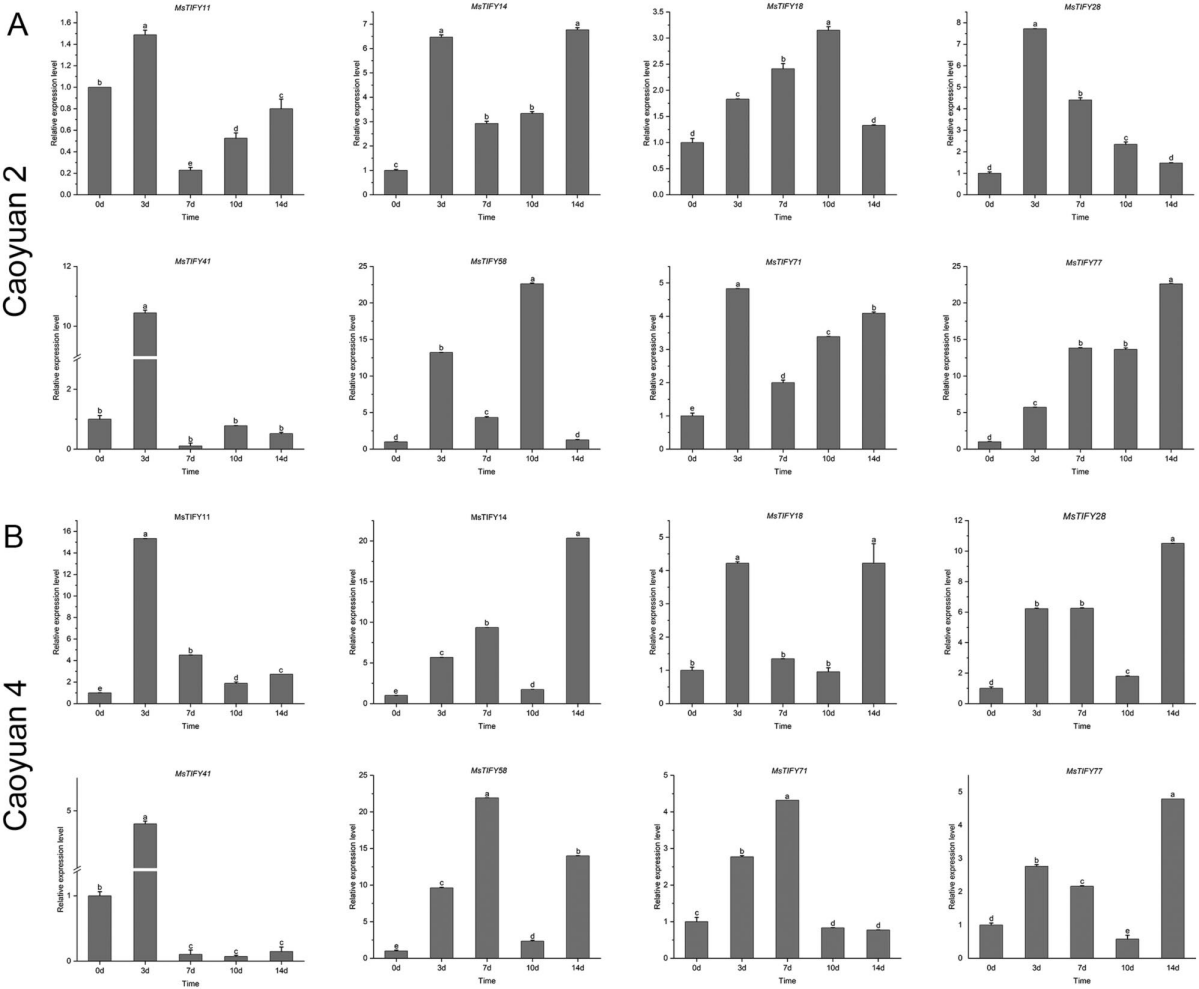
qRT-PCR analysis of expression patterns of eight *MsTIFY* genes under thrips stress of ‘Caoyuan No.2’ and ‘Caoyuan No.4’. **A**: The expression patterns of eight *MsTIFY* genes under thrips stress of Caoyuan No.2. **B**: The expression patterns of eight *MsTIFY* genes under the stress of Caoyuan No.4. The error bars indicate the standard errors of three biological replicates. Columns with different letters are significantly difference(*P* < 0.05)

To clarify the expression patterns of the alfalfa *TIFY* gene family under biotic stresses, we analyzed the expression levels of 8 genes (*MsTIFY11*, *MsTIFY14*, *MsTIFY18*, *MsTIFY28*, *MsTIFY41*, *MsTIFY58*, *MsTIFY71* and *MsTIFY77*) in two alfalfa varieties, ‘Caoyuan No.2’ (a thrips-sensitive variety) and ‘Caoyuan No.4’ (a thrips-resistant variety), at different times (0d, 3d, 7d, 10d and 14d) with thrips infection (Fig. 5; Tab.S5).

The results showed that all 8 *MsTIFY* genes were induced by the thrips infection in 3d in both ‘Caoyuan No.2’ and ‘Caoyuan No.4’, suggesting that these genes may play essential roles in the early response to biotic stress in alfalfa (Fig. 5A, B). In detail, *MsTIFY11* was significantly up-regulated only in the 3d thrips infection but downregulated in 7d, 10d, and 14d thrips infection in both varieties. In addition, *MsTIFY14* and *MsTIFY77* were highly induced by 14d thrips infection in both varieties. These results suggested that different *MsTIFY* genes play roles in various stages of plant-insect interactions.

In Caoyuan No.2, *MsTIFY28* reached the highest level after 3d of thrips infection, and then it gradually decreased. *MsTIFY18* and *MsTIFY58* showed significant up-regulation after 10d of thrips infection, while the expression of *MsTIFY77* showed an upward trend. Interestingly, *MsTIFY11*, *MsTIFY14*, and *MsTIFY71* showed the highest expression levels on the 3d of thrips infection, which then rapidly decreased and gradually increased again (Fig. 5A). The expression patterns of *MsTIFYs* in ‘Caoyuan No.4’ were similar to those of ‘Caoyuan No.2’ (Fig. 5B).

### Expression of *MsTIFY* genes in response to abiotic stress

**Fig. 6:**
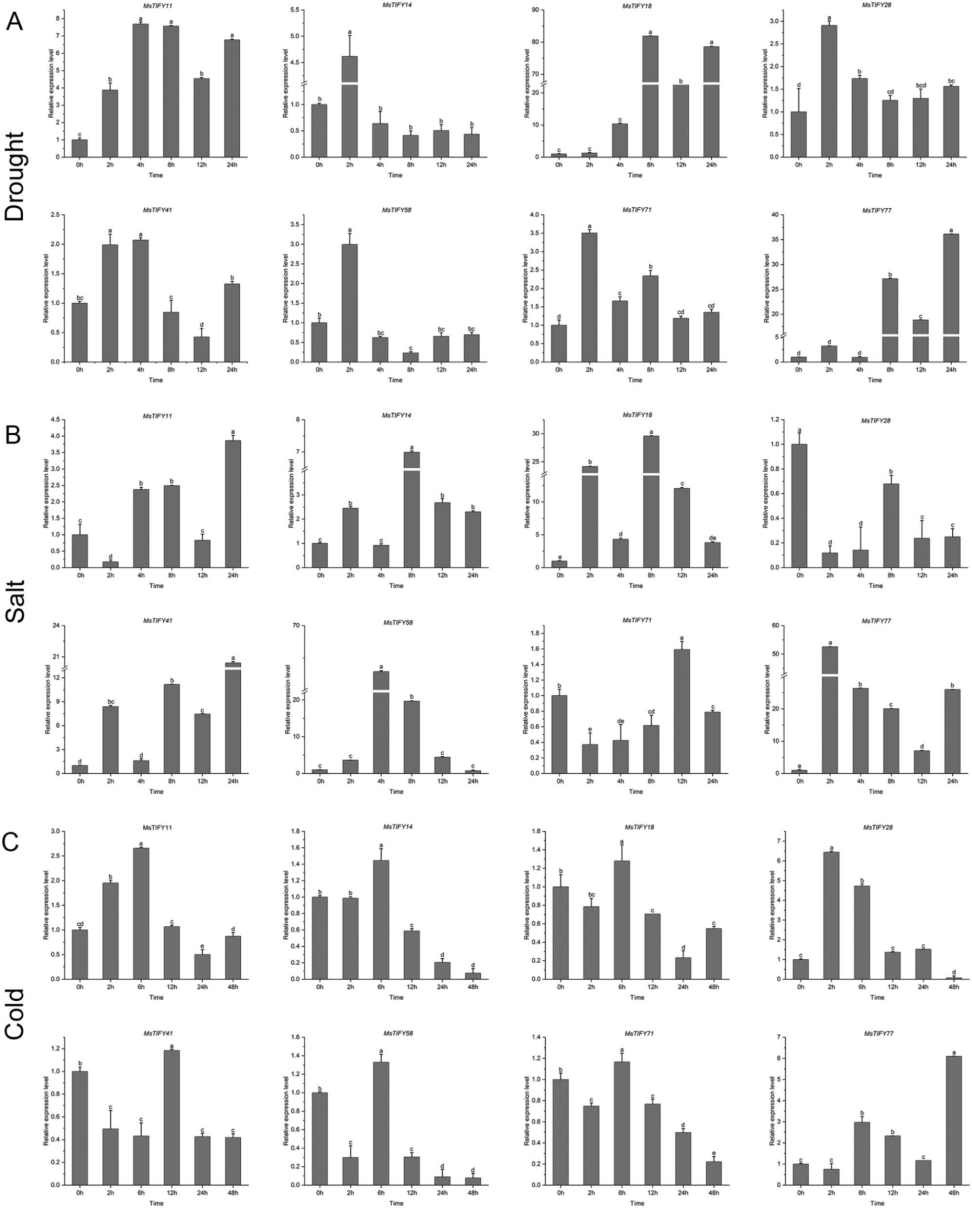
qRT-PCR analysis of expression patterns of eight *MsTIFY* genes under different abiotic stresses. **A**: Drought; **B**: Salt; **C**: Cold. The error bars indicate the standard errors of three biological replicates. Columns with different letters are significantly difference(*P* < 0.05)

To explore the potential functions of the *MsTIFY* genes in response to abiotic stress, the expression of 8 screened *MsTIFY* genes in response to drought, salt, and cold in ‘Caoyuan No.4’ were analyzed (Fig. 6; Tab.S6).

The results showed that all the *MsTIFYs genes were* up-regulated under different degrees of drought stress (Fig. 6A). In detail, a significant up-regulation of the *MsTIFY11* expression was observed at different status of drought stress by up to 3.87- to 7.69- fold of the control. For *MsTIFY14*, *MsTIFY28*, *MsTIFY41*, *MsTIFY58*, and *MsTIFY71*, the expression level significantly increased at 2 h and then declined and maintained the control level. In addition, transcripts of *MsTIFY18* and *MsTIFY77* increased in the later stage of drought stress by up to 81.89- and 36-fold of the control, respectively.

Under salt stress, the expression level of the majority of *MsTIFYs* genes was fluctuation rising, including *MsTIFY11*, *MsTIFT14*, *MsTIFY18*, *MsTIFY41* and *MsTIFY77* (Fig. 6B). For *MsTIFY28*, the gene expression was down to 0.12 to 0.68 folds of the control. The expression of *MsTIFY71* clearly declined except at 12 h, and *MsTIFY58* expression increased before 4 h and then gradually decreased.

The results showed that *MsTIFY* genes presented different expression patterns under cold treatment at 4℃ (Fig. 6C). In detail, the expression of *MsTIFY77* was significantly up-regulated of the control. The expression of *MsTIFY11*, *MsTIFY14*, *MsTIFY18*, and *MsTIFY28* gradually increased in the earlier stage of cold treatment but progressively declined with the extension of treatment time. The expression of *MsTIFY58* and *MsTIFY71* were significantly lower than those of control except at 6 h. Interestingly, the expression level of *MsTIFY41* clearly declined to about half of the control except at 12 h.

### Expression of *MsTIFY* genes in response to plant hormones

**Fig. 7:**
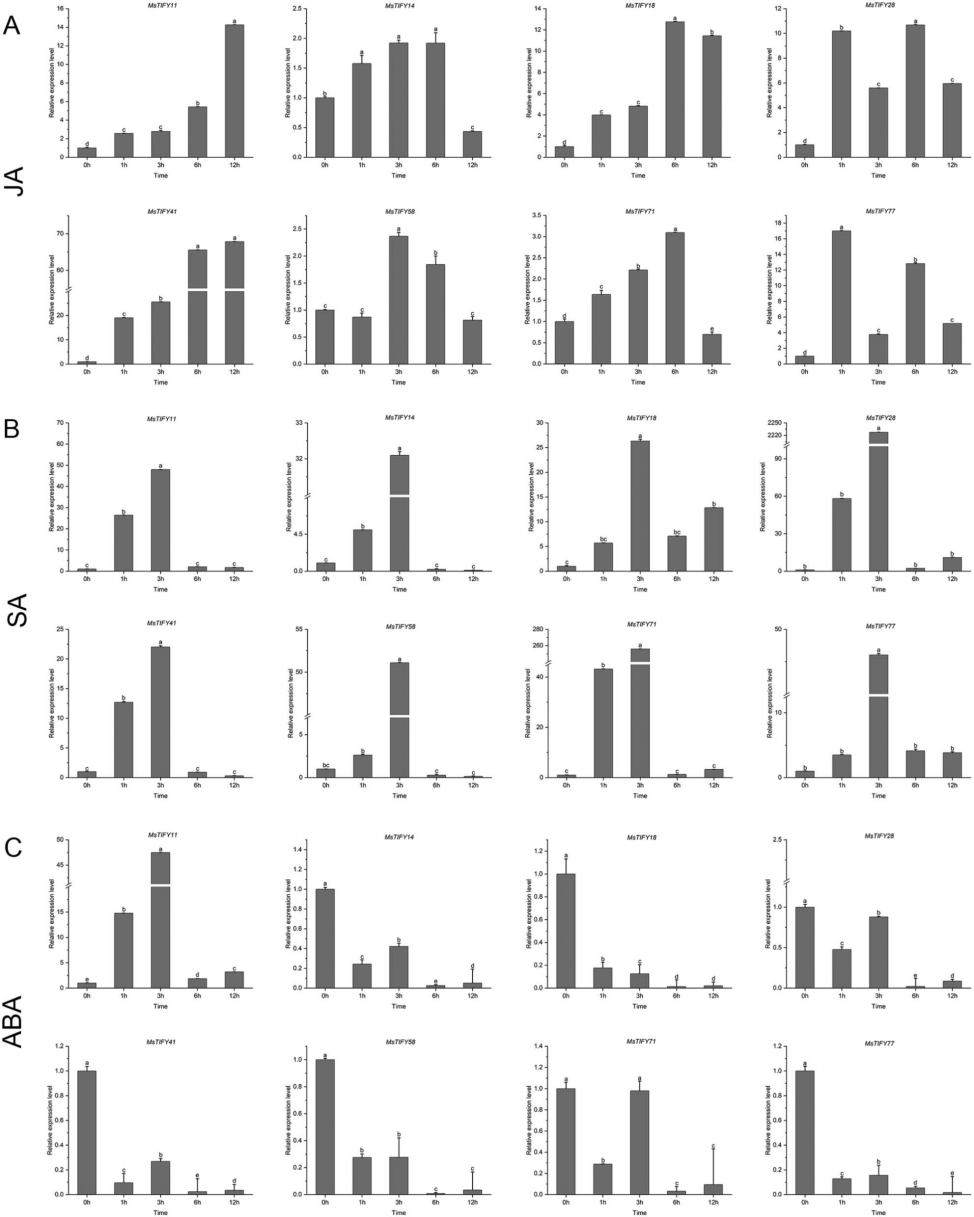
qRT-PCR analysis of expression patterns of 8 *MsTIFY* genes under different hormone stresses. **A**: JA; **B**: SA; **C**: ABA. The error bars indicate the standard errors of three biological replicates. Columns with different letters are significantly difference (*P* < 0.05)

Hormones are essential factors affecting plant growth and development [27, 28]. The expression levels of 8 genes in response to JA, SA, and ABA treatments in ‘Caoyuan No.4’ were analyzed by qRT-PCR (Fig. 7; Tab.S7).

The results showed that all *MsTIFY* genes were up-regulated at different times after spraying JA (Fig. 7a). In detail, the expression levels of *MsTIFY11* and *MsTIFY41* were gradually increased. *MsTIFY14*, *MsTIFY1*8, *MsTIFY58*, and *MsTIFY71* had similar expression patterns, which gradually increased or maintained in the earlier stage while declining at 12 h. In addition, the *MsTIFY28* and *MsTIFY77* expression levels fluctuated and rose.

For SA treatment, all *MsTIFY* genes showed a similar expression pattern (Fig. 7b), which gradually increased at 3 h and decreased sharply at 6–8 h and then up to the control level except *MsTIFY18*.

The results showed that all the *MsTIFYs* genes decreased at different times under ABA treatment except *MsTIFY11* (Fig. 7C). In detail, the expression of *MsTIFY14*, *MsTIFY18*, *MsTIFY28*, *MsTIFY41*, *MsTIFY58*, and *MsTIFY77* were clearly declined to about 0.2- to 0.4- fold of the control. In contrast to these genes, a significant upregulation of *MsTIFY11* was observed at any treatment time and reached 47.48- fold of the control at 3 h.

**Fig. 8:**
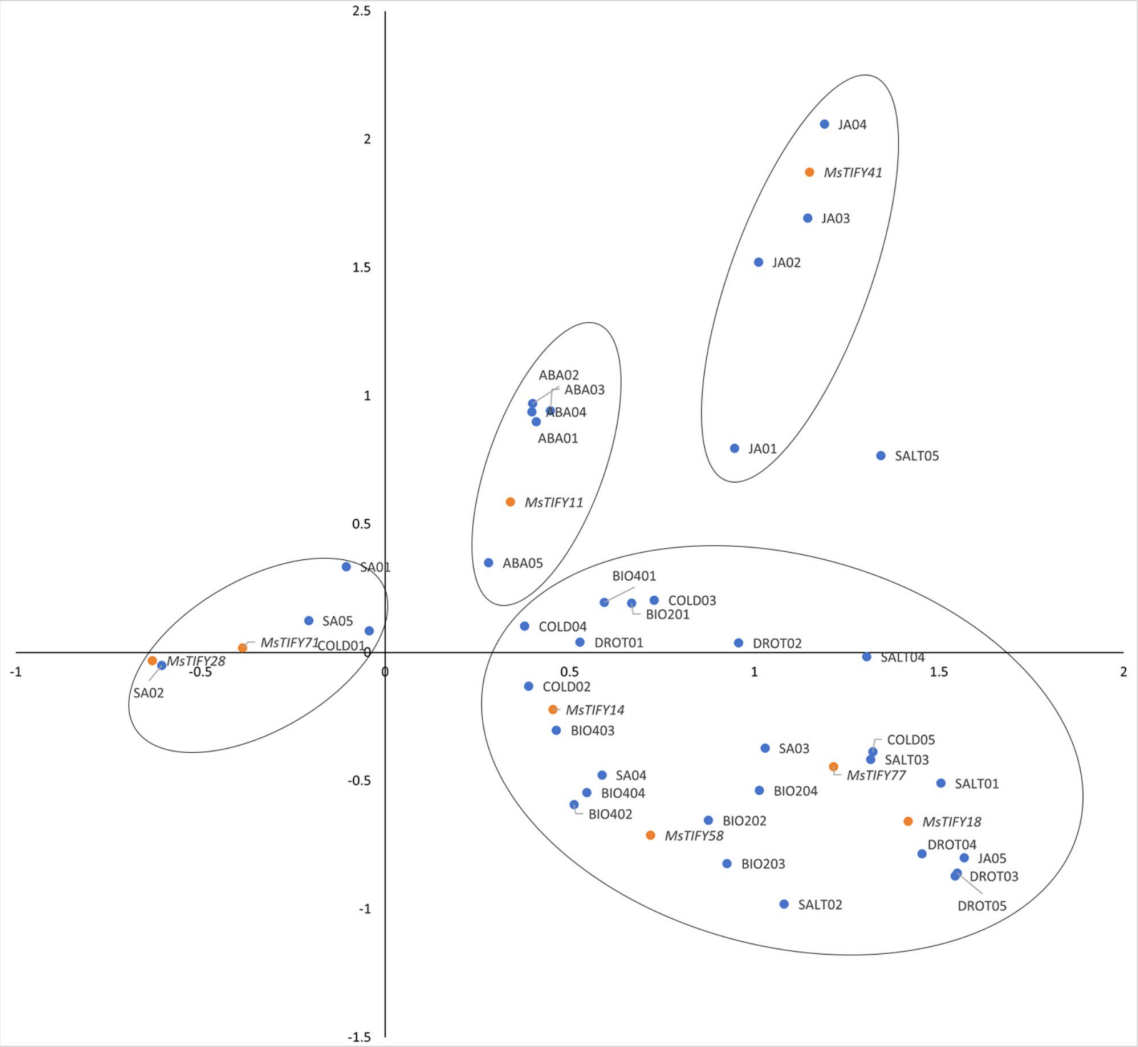
Correspondence analysis of 8 *MsTIFYs* with different stresses. BIO2: Thrips stress of Caoyuan No.2; BIO4: Thrips stress of Caoyuan No.4; DROT: Drought stress; SALT: Salt Stress; COLD: Cold stress; JA: JA stress; SA: SA stress; ABA: ABA stress

To further clarify the response of the 8 *MsTIFYs* genes to different treatments, correspondence analysis was performed (Fig. 8). The results showed that *MsTIFY41* was more closely related to JA, *MsTIFY11* was more closely related to ABA, and *MsTIFY28* and *MsTIFY71* were more closely related to SA. Furthermore, *MsTIFY14* was closer to thrips stress and cold stress, while *MsTIFY58* was mainly closer to thrips stress. In addition, *MsTIFY18* and *MsTIFY77* were closer to the late stage of drought, salt and cold stress *MsTIFY14*, *MsTIFY18*, *MsTIFY58* and *MsTIFY77* had closer correspondences to thrips, drought, salt, and cold stress.

## Discussion

The *TIFY* gene family is unique in plants [29]. In the study on the *TIFY* gene family, the number of *TIFY* genes in most plants was not more than 30, such as Arabidopsis with 18 [11], rice with 20 [18], Terrestris with 21 [30], cotton with 21 [31], Moso bamboo with 24 [32], tomato with 26 [33], kiwifruit with 27 [34] and peanut with 29 [35]. We identified 84 *TIFY* genes in alfalfa, the largest number of *TIFY* family members studied so far, which might be caused by the doubling of the alfalfa genome. In addition, different research methods may also lead to differences in the numbers identified. For instance, 30 *TIFY* genes were identified before [36], but 47 were identified in the latest study [17]. Wheat had 49 [20] and then 63 [37]. As a result, newer methods can identify more gene family members. The analysis of family members showed that among the *TIFY* gene family, the JAZ subfamily had the most family members, while the TIFY and PPD families had fewer family members. For example, among the 15 *TIFY* gene family members in watermelon, there were 8 *CIJAZs*, 4 *CIZMLs*, 2 *CITIFYs*, and 1 *ClPPDs* [38]. Among the 12 *TIFY* gene families in birch, there were 7 *BpJAZs*, 3 *BpZMLs*, 1 *BpTIFYs*, and 1 *BpPPDs* [39]. We identified 84 *TIFY* genes in alfalfa (Table 1), including 58 *MsJAZs*, 18 *MsZMLs*, 4 *MsTIFYs*, and 4 *MsPPDs*, which were similar to those found in other species. In addition, TIFY family genes were found in bryophytes, stonecrops, monocotyledons, and dicotyledons (Table 1; Fig. 1)., but within each subfamily, plants originating from the same phylum have closer *TIFY* evolutionary relationships. For example, dicotyledons are more clustered on one branch of the *TIFY* evolutionary relationship. This suggests that the genes of the dicotyledonous *TIFY* family evolved later than those of the bryophyte phylum, the lithophyte phylum, and the monocotyledonous phylum.

*TIFY* family genes had diverse structural domains. In this study, TIFY, JAS/CCT-2, CCT and GATA were found in the TIFY proteins (Tab.S8). The motif structure in each TIFY subfamily had mostly conservative domains. The *MsTIFY* gene family was classified into four subfamilies (TIFY, JAZ, ZML, and PPD) according to the characteristics of its conserved domain, and the JAZ subfamily could be further divided into five subgroups. This is consistent with previous studies [39]. *AtTIFY8* interacts with the transcription factor REVOLUTA of HD-ZIP III and regulates leaf senescence [40]. In this study, *MsTIFY50*, *MsTIFY55*, *MsTIFY56*, and *MsTIFY57* cluster into the same clade, suggesting that they may have similar functions (Fig. 2). *OsTIFY11b* can increase carbohydrate accumulation in stem and leaf sheath [19], regulate the growth and development of stem and leaf [14], and improve salt tolerance [20]. In our study, *OsTIFY11b* was clustered into the same branch with 38 *MsTIFYs*, among which it was close to *MsTIFY3*, *MsTIFY4*, *MsTIFY11*, *MsTIFY12*, and *MsTIFY21*, was predicted to have similar functions (Fig. 1). In addition, qRT-PCR also found that *MsTIFY11* was highly expressed in roots, stems and leaves (Fig. 4), and was significantly up-regulated after salt stress (Fig. 5), so it is speculated that *MSTIFY11* plays a vital role in regulating stem and leaf growth and salt stress tolerance.

*MsTIFY* family genes have tissue specificity. The expression profiles of 8 *MsTIFYs* were identified in 7 different tissues (roots, stems, old leaves, tender leaves, flowers, pods and seeds) (Fig. 4). The results showed that most *MsTIFYs* were highly expressed in roots, especially *MsTIFY14*, *MsTIFY71*, *MsTIFY18*, *MsTIFY11* and *MsTIFY58*, suggesting that *TIFY* gene family may participate root growth and development. *MsTIFY77* was expressed preferentially in stems, and *MsTIFY28* was expressed highest in young leaves. *MsTIFY41* was expressed preferentially in old leaves and was highly expressed in roots and stems. Interestingly, *OsTIFY1a* and *OsTIFY1b* are highly expressed in leaves [14], which are in the same clade as *MsTIFY41* in the evolutionary tree (Fig. 2). In rice [19], most *OsTIFY* genes are mainly expressed in leaves. *CmJAZ* delays the flowering of chrysanthemums. In Astragalus [41], JAZ proteins interact with haemoglobin via TIFY domains and are involved in nodal development and nitrogen fixation.

*MsTIFY* family genes play an essential role in biotic stresses. The expression of the same gene was different in different alfalfa varieties. In sorghum [42], after JA treatment and aphid infection, the expression of *SbJAZ1*, *SbJAZ5*, *SbJAZ13*, and *SbJAZ16* in resistant varieties was up-regulated. In apples [43], the expression level of *MsTIFY10B-a* and *MsTIFY9-c* in resistant strains increased 34 times and 5.2 times after insect infestation. In maize [44], *ZmJAZ1* and *ZmCOI1a* responded to the feeding of autumn armyworms (*Spodoptera frugiperda*). In the studies on the response of *the TIFY gene family to biological stress*,* there are only a few studies on infectious pathogens* and almost no studies on butt worms. In cucumber [45], *CsJAZ1* and *CsJAZ2* showed significant changes in infection with four diseases (powdery mildew, downy mildew, stem blight, and grey mould). In tea plants [46], Colletotrichum camelliae mainly up-regulated the expression levels of *CsJAZ1* and *CsJAZ10*. In this study, thrips susceptible and thrips resistant alfalfa varieties with different various degrees of thrips damage were selected to observe the expression changes of 8 *MsTIFYs* genes (Fig. 5). The results showed that 8 *MsTIFYs* were significantly up-regulated in both varieties at 3d of thrips feeding, indicating that they could positively regulate the early defense of alfalfa against thrips. *MsTIFY14* and *MsTIFY77* were also significantly up-regulated in the expression of thrips on the 7d and 14d of thrips feeding, suggesting that they may have an important role in alfalfa defense against thrips at later stages.

A growing number of studies have shown that *TIFY* transcription factors play an important regulatory role in abiotic stresses [47–50]. In wheat [20], salt treatment induced the expression of *TdTIFY11*. In bamboo [32], 50% of *PeTIFY* genes could be up-regulated by dehydration stress. In soybean [16], salt stress induced *GmTIFY10e* and *GmTIFY10g*. In rice [14], overexpression of *OsTIFY11a* increased tolerance to salt and dehydration stress. In our study, most *MsTIFYs* were up-regulated by drought, salt, and cold stress (Fig. 6). Drought significantly induced *MsTIFY11*, *MsTIFY18*, *MsTIFY28*, *MsTIFY71*, and *MsTIFY77*. Salt stress significantly induced *MsTIFY18*, *MsTIFY41*, and *MsTIFY77*, and cold significantly induced *MsTIFY28* and *MsTIFY77*. In watermelon [38], JA activated 8 genes and inhibited 1 gene, among which *CIJAZ1* and *CIJAZ7* were most significantly induced. In tomato [33], *SlJAZ1*, *SlJAZ*3, *SlJAZ*6, *SlJAZ*7 and *SlJAZ*11 were significantly induced by JA. Although ABA could induce some genes, their expression levels were not as high as those of JA. However, in grapes [51], many of the TIFY genes were responsive to JA and ABA but not SA or ET. In our study, it was also found that almost all *MsTIFYs* were induced by JA and SA, and ABA significantly inhibited the expression of most *MsTIFYs* (Fig. 7). Therefore, we speculated that JA and SA could induce the expression of *MsTIFY* family genes. In contrast, ABA could inhibit most of them. Therefore, the results indicate that *MsTIFY* genes actively respond to biotic and abiotic stresses.

Further, the correspondence analysis of 8 genes with the expression of genes after different stresses revealed that *MsTIFY58* was mainly related to insect stress, and *MsTIFY18* and *MsTIFY77* were mainly involved in the late stage of drought, salt and cold stresses. *MsTIFY58* may be important for responding to insect stress. *MsTIFY14* was in response to both thrips stress and cold stress. In addition, presumably based on correspondence analysis *MsTIFY41 and MsTIFY11* may play more dominant roles mainly in regulating JA and ABA, respectively. *MsTIFY28 and MsTIFY71 genes may primarily play an important role in the response to SA* and early cold stress.

## Materials and methods

### Identification of the *TIFY* gene family in alfalfa

To identify more comprehensive TIFY family genes, the sequence of *Physcomitrella patens* of *Bryophyta*, *Selaginella moellendorffii* of *Lycopodiophyta*, *Oryza sativa* of *Monocots*, *Arabidopsis thaliana* and *Medicago truncatula* in *Eudicots* was downloaded from the Ensemblplants database (http://plants.ensembl.org/). However, we didn’t find a genome database of alfalfa in the Ensemblplants database. Thus, the protein sequence and corresponding CDS sequence of alfalfa were obtained from Alfalfa Breeder’s Toolbox database (https://alfalfatoolbox.org/) by homologous sequence alignments.

The sequence characteristics of the TIFY domain (PF06200) were searched in the Pfam database (http://pfam.xfam.org/), and the HMM file was formed as a reference file for conservative domain comparison [52]. Then, according to Bai [12], the HMMER program was used to compare the protein sequence data in the above genomic data respectively and screen E-value > 1e-6, the amino acid sequence with a score higher than 20 [53, 54]. The BLASTP program was then used to compare these sequences to *Arabidana* seed sequences. Proteins containing the TIFY domain were separated into different subfamilies based on the presence or absence of TIFY, PPD, CCT-2, CCT, or ZML domains by removing duplications, incorrect sequencing, incomplete read frames, or incomplete domain sequences.

### Conserved motif and phylogenetic analysis of *MsTIFY* proteins

To explore the phylogenetic relationship of *MsTIFY* transcription factors, the molecular evolutionary relationship of the *MsTIFY* gene family was elucidated by constructing an evolutionary tree. The sequences were aligned with MAFFT using the ‘auto’ strategy and normal alignment mode [55]. Gap sites were removed with trimAl using “noallgaps” command [56]. The best-fit model for 178 amino acid sequences of 6 different plants was JTT + R5. The best-fit model for the *MsTIFY* family in *Medicago sativa was* JTT + G4. Maximum likelihood phylogenies were inferred using IQ-TREE under the JTT + R5 or JTT + G4 model for 20,000 ultrafast bootstraps, as well as the Shimodaira–Hasegawa–like approximate likelihood-ratio test [57–59]. The conserved motif of *MsTIFY* was determined using MEME online analytical tools (https://meme-suite.org/meme/tools/meme). In addition, NCBI Conserved Domains were used to analyze the Conserved domain of alfalfa TIFY family amino acid sequence [60].

### Protein interaction network diagram prediction for MsTIFYs

Cystoscope version 3.7.0 was used to search for the interaction factors of these proteins to construct PPI networks for the MsTIFY gene family and related families. The search was conducted on the STRING database (https://string-db.org/) and the organism selected was *Arabidopsis thaliana*. These proteins were then mapped to the MsTIFY family in *Medicago sativa* with the following cutoff values: identity ≥ 30 and E-value ≤ 1E-10. If the value exceeds this value, it will not be displayed. Then, the single gene with the highest hit ratio (lowest E value) was selected to represent each protein in the network, and the related protein regulatory network was established.

### Plant materials and stress treatments

Alfalfa seeds (Caoyuan No.4 and Caoyuan No.2) were bred at Inner Mongolia Agricultural University, China. All plants were cultivated in pots (H10 cm × D12 cm, one plant per pot) containing field-collected soil in a greenhouse with a relative humidity of 60 ± 5% and 70 ± 5% at 30 ± 5 °C and 20 ± 5 °C during day and night, respectively. Plants were watered every other day.

Thrips infections were treated as described by Tu et al. [61]. with some modifications. When the seedlings were about 40 days old, they were randomly and equally divided into two groups: (1) 100 thrips per plant were placed onto the leaves and covered by a cage with 300-mesh nylon cloth, and (2) In order to avoid the interference of growth conditions on the test results, this test was sampled at the same time (at the same time at 54 days of growth), and thrips injection was carried out 14, 10, 7, 3 and 1 days before sampling, respectively, and alfalfa that was not inoculated with thrips on the day of sampling was used as a control. For salt and drought stresses, the seedlings (Caoyuan No.4) were treated with 250mmol/L NaCl and air drought [62] and harvested at 0 h, 2 h, 4 h, 8 h, 12 h, and 24 h. For cold stress, the seedlings were placed in a low-temperature incubator at four °C, and samples were collected at 0 h, 2 h, 6 h, 12 h, 24 h, and 48 h. In addition, for hormone treatments, including salicylic acid (SA), abscisic acid (ABA), and jasmonic acid (JA) treatments, 0.5mmol/L SA, 10umol/LABA, and 100umol/L MeJA were sprayed on the plants, to minimize errors, spray each plant five times, then seal it with a transparent plastic bag to prevent hormone volatilization, and harvest at the same time at 0 h, one h, 3 h, 6 h and 12 h. Samples of roots, stems, mature leaves, young leaves, flowers, seeds, and pods of alfalfa were collected for tissue-specific expression analysis. All samples were set in triplicate. Immediately after sampling, the samples were snap-frozen in liquid nitrogen and stored at -80 °C for subsequent analysis.

### Quantitative real-time PCR analysis

Eight *MsTIFYs* were screened for qRT-PCR experiments. The total RNA of different samples was extracted using OminiPlant RNA Kit (Cwbio, Beijing, China) according to the manufacturer’s instructions. RNA concentration and purity were measured by a NanoDrop ND1000 spectrophotometer. Total RNA was used for first-strand cDNA synthesis using EasyQuick RT MasterMix (Cwbio, Beijing, China). The synthesized cDNA was used as a template for gene expression analysis. The qRT-PCR experiments were performed with 2×MagicSYBR Misture on a 7500 Real-time PCR system (Applied Biosystems, Foster City, CA, USA), using the alfalfa β-actin gene as a reference gene. NCBI Primer-BLAST (https://blast.ncbi.nlm.nih.gov/Blast.cgi) was used to design primers (Tab.S8). Three independent biological replicates and two technical replicates for each sample were used for the qRT-PCR. The gene expression was quantified by the 2^−ΔΔCT^ method [63].

### Statistical analysis

Excel 2010 was used for the statistics calculation of relevant data, SPSS Statistics 18.0 software was used for the analysis of variance, and a student t-test was used to compare the mean values at a 5% significance level. Use the Origin 2019 software to make bar charts. SAS was used to analyze 8 genes in correspondence with their expression after different stresses.

## Conclusion

In this study, a total of 84 *TIFY* genes were identified in the alfalfa, including 58 *MsJAZs*, 18 *MsZMLs*, 4 *MsTIFYs*, and 4 *MsPPDs*. qRT-PCR data of 8 random selected genes in different tissues, biotic and abiotic stress revealed that most of *MsTIFY* genes were highly expressed in roots and leaves. All 8 *MsTIFY genes* were significantly induced by early thrips feeding in the resistant and susceptible alfalfa varieties, *MsTIFY14* and *MsTIFY77* may be concurrently involved in alfalfa defense against thrips in the late stages. Different abiotic stresses, including drought, salt, and cold, could induce or inhibit the expression of 8 *MsTIFY* genes to varying degrees. In addition, *MsTIFY genes* were significantly up-regulated by JA and SA. Interestingly, *MsTIFY77 was induced considerably* by all the biotic, abiotic, or plant hormones (JA or SA) except ABA. Our results laid the foundation for further study for further study on molecular functions of *TIFYs* in alfalfa.

## Electronic supplementary material

Below is the link to the electronic supplementary material.

Supplementary Material 1

Supplementary Material 2

Supplementary Material 3

Supplementary Material 4

Supplementary Material 5

Supplementary Material 6

Supplementary Material 7

Supplementary Material 8

Supplementary Material 9

## Data Availability

All data generated or analyzed during this study are included in this published article [and its supplementary information files]. The datasets supporting our conclusions regarding the current study are included in the manuscript and the additional file. We did not generate any sequencing data in this study. We have used already published genomic data. The sequence of *Physcomitrella patens* of *Bryophyta*, *Selaginella moellendorffii* of *Lycopodiophyta*, *Oryza sativa* of *Monocots*, *Arabidopsis thaliana*, and *Medicago truncatula* in *Eudicots* was downloaded from the Ensembl plants database (http://plants.ensembl.org/). The protein sequence and corresponding CDS sequence of alfalfa were obtained from Alfalfa Breeder’s Toolbox database (https://alfalfatoolbox.org/).
