# Supplementary material for: Genome-wide investigation of the TIFY transcription factors in alfalfa (Medicago sativa L.): identification, analysis, and expression
Source: BMC Plant Biol. 2024 Sep 6;24:840. doi: 10.1186/s12870-024-05378-w (PMC11378388; doi:10.1186/s12870-024-05378-w)
Supplement: Supplementary file 6 — Supplementary Material 6 [file 12870_2024_5378_MOESM6_ESM.docx]

**Supplementary Information**

Table S*1: List of the TIFY family genes in Arabidopsis thaliana, Oryza sativa, Physcomitrella patens, Selaginella moellendorffii*, *Medicago trunculata* and *Medicago sativa.*

Table S2: Summary of the structure of the *TIFY* family genes in alfalfa.

Table S3: Protein interaction network Statistics.

Table S4: Premier sequences used in qRT -PCR.

Table S5: Expression of *MsTIFY* genes in different tissues.

Table S6: Expression of *MsTIFY* genes in response to thrips feeding.

Table S7: Expression of *MsTIFY* genes under drought, salt and cold stresses.

Table S8: Expression of *MsTIFY* genes under hormones stresses.
